# Investigation of IgG4‐positive cells in idiopathic multicentric Castleman disease and validation of the 2020 exclusion criteria for IgG4‐related disease

**DOI:** 10.1111/pin.13185

**Published:** 2021-11-11

**Authors:** Asami Nishikori, Midori Filiz Nishimura, Yoshito Nishimura, Kenji Notohara, Akira Satou, Masafumi Moriyama, Seiji Nakamura, Yasuharu Sato

**Affiliations:** ^1^ Division of Pathophysiology Okayama University Graduate School of Health Sciences Okayama Japan; ^2^ The Research Program for Intractable Disease by Ministry of Health, Labor and Welfare Japanese Pathology Study Group of IgG4‐Related Disease Tokyo Japan; ^3^ Department of Pathology Okayama University Graduate School of Medicine, Dentistry, and Pharmaceutical Sciences Okayama Japan; ^4^ Department of General Medicine Okayama University Graduate School of Medicine, Dentistry, and Pharmaceutical Sciences Okayama Japan; ^5^ Department of Medicine, John A. Burns School of Medicine University of Hawai'i Honolulu Hawaii USA; ^6^ Department of Anatomic Pathology Kurashiki Central Hospital Okayama Japan; ^7^ Department of Surgical Pathology Aichi Medical University Hospital Aichi Japan; ^8^ Section of Oral and Maxillofacial Oncology, Division of Maxillofacial Diagnostic and Surgical Sciences, Faculty of Dental Science Kyushu University Fukuoka Japan

**Keywords:** exclusion criteria, idiopathic multicentric Castleman disease, IgG4‐related disease, lung, lymph node

## Abstract

Patients with plasma cell type idiopathic multicentric Castleman disease (PC‐iMCD) often show elevated serum IgG4 levels and IgG4‐positive cell infiltration in tissues due to overproduction of interleukin‐6, and may meet the diagnostic criteria for IgG4‐related disease (IgG4‐RD). Although PC‐iMCD has been listed as a major exclusion disease for IgG4‐RD, distinguishing between these diseases is challenging due to a lack of highly specific diagnostic biomarkers. In 2020, we proposed exclusion criteria of IgG4‐RD mimickers. In this paper, we validated the accuracy of the criteria in excluding one of the mimickers, PC‐iMCD, from IgG4‐RD. Validation was performed on 57 PC‐iMCD patients (39 presenting lymph node lesions and 19 with lung lesions) and 29 IgG4‐RD patients (22 presenting lymph node lesions and seven with lung lesions). According to our results, 20.5% of the PC‐iMCD patients with lymph node lesions and 42.1% of those with lung lesions met the diagnostic criteria for IgG4‐RD. All these patients with PC‐iMCD were excluded from a diagnosis of IgG4‐RD by the proposed criteria. Additionally, 6.9% of IgG4‐RD patients met the exclusion criteria. Thus, if the exclusion criteria are met, diagnosis should be made based on a combination of findings including organ distribution of disease, response to steroid therapy, and other pathological findings.

**Abbreviations**
CRP: C‐reactive protein
IgG4‐RD: immunoglobulin G4‐related disease
IL‐6: interleukin‐6
PC‐iMCD: plasma cell type idiopathic multicentric Castleman disease

## INTRODUCTION

Castleman disease is a rare lymphoproliferative disorder that shares several basic pathological features. Castleman disease is classified into multicentric and unicentric types based on its clinical manifestations.^1^ In multicentric Castleman disease (MCD), patients typically present with lymphadenopathy that involves more than one lymph node region. MCD is divided according to Kaposi sarcoma‐associated herpesvirus/Human herpesvirus type 8 (KSHV/HHV8) infection status, and idiopathic MCD (iMCD) is defined as the group of KSHV/HHV8‐negative MCD excluding those seen in association with POEMS syndrome (polyneuropathy, organomegaly, endocrinopathy, M‐proteins, and skin changes). Further, iMCD is pathologically sub‐classified into plasma cell (PC) and hypervascular (HV) types, with the former being predominant.^2^, ^3^ In PC type idiopathic multicentric Castleman disease (PC‐iMCD), overproduction of interleukin‐6 (IL‐6) is deeply involved in the pathogenesis of the disease, which leads to systemic symptoms, such as fever, elevated acute‐phase protein and hypergammaglobulinemia in blood, and sheet‐like PC proliferation in tissues.^3^, ^4^

IgG4‐related disease (IgG4‐RD) is a systemic disorder characterized by high serum IgG4 levels and mass‐forming lesions with fibrosis and infiltration of abundant IgG4‐positive cells.^5^, ^6^ The disease affects various organs, including the pancreas, salivary glands, lacrimal glands, lymph nodes, and lungs. IgG4‐RD‐associated lymph node lesions are pathologically classified into five types,^6^, ^7^ and cases with generalized lymphadenopathy often show a pathological pattern similar to that of PC‐iMCD.

A major problem in the diagnosis of IgG4‐RD is the difficulty in differentiating it from its mimickers. Hyper IL‐6 syndrome, including PC‐iMCD, is one of the most important mimickers. Hyper IL‐6 syndrome is associated with polyclonal hypergammaglobulinemia, accompanied by elevated serum IgG4 levels and tissue infiltration of IgG4‐positive cells. PC‐iMCD is mentioned as an exclusion disease in the diagnostic criteria for IgG4‐RD, but in practice, it is difficult to differentiate between the two because of overlapping features. However, the treatment strategy is different; IgG4‐RD is typically responsive to steroid monotherapy, whereas PC‐iMCD is less responsive to steroids as first‐line therapy, and thus IL‐6 receptor blockade therapy is required.^8^, ^9^ Therefore, accurate differentiation of IgG4‐RD mimickers, such as PC‐iMCD, is important to avoiding misdiagnosis and providing incorrect treatments. In 2020, the Japanese Pathology Study Group of IgG4‐related Disease (Tokyo, Japan) proposed exclusion criteria to distinguish these mimickers (Table 1),^10^ but no validation studies have been performed.

**Table 1:** The 2020 exclusion criteria for IgG4‐related disease

Clinical findings
Continuing elevated serum level of CRP (≧1.0 mg/dL)^a^ Elevated serum level of IgA^b^ Elevated serum level of IgM^b^
**Pathological findings**
Sheet‐like proliferation pattern of mature plasma cells
High degree of hemosiderin deposition
Neutrophilic infiltration

*Note*: Cases that are present with any one of the clinical or pathological findings listed below cannot be categorized as definite IgG4‐RD, although they may meet the diagnostic criteria for IgG4‐RD.

^a^ Continuing elevation of uncertain cause.

^b^ Serum level above normal range is defined as “elevated.” Reference value of each institution should be applied.

Ref. 10.

In this study, we assessed whether the proposed exclusion criteria of IgG4‐RD^10^ could effectively exclude PC‐iMCD using lymph node and lung materials. We also examined the number of IgG4‐positive cells in PC‐iMCD and discussed the relationship between the number of IgG4‐positive cells and serum immunoglobulin levels.

## MATERIALS AND METHODS

### Patient selection

In total, 86 Japanese patients with PC‐iMCD (*n* = 57) and IgG4‐RD (*n* = 29) between 1996 and 2021 at the Department of Pathology (Okayama University, Japan) were pathologically examined. In PC‐iMCD, 39 cases presented lymph node lesions and 19 cases exhibited lung lesions. One patient had both lymph node and lung lesions. In IgG4‐RD, 22 cases presented lymph node lesions and seven cases presented lung lesions. The lung lesions in both PC‐iMCD and IgG4‐RD are the same as those included in our previous report.^11^ Patients with autoimmune/rheumatic disorders, infections, and lymphomas were clinically and pathologically excluded.

All IgG4‐RD patients met both the comprehensive diagnostic criteria for IgG4‐RD^12^ as well as pathological criteria (IgG4‐positive cells >50/high‐power fields [HPFs] and IgG4/IgG‐positive cell ratio >40%).^5^

All PC‐iMCD patients with lymph node lesions met the consensus diagnostic criteria for iMCD.^3^ PC‐iMCD patients with lung lesions were diagnosed by a combination of laboratory and clinical criteria of the consensus diagnostic criteria for iMCD^3^ and the presence of enlarged lymph nodes in ≥2 lymph node stations. The study protocol was approved by the institutional review board of Okayama University (Okayama, Japan).

### Pathological examination and immunohistochemistry

All lymph node and lung specimens were fixed in 10% formaldehyde and embedded in paraffin. These paraffin‐embedded tissue blocks were sliced into 3 µm thick sections, which were stained with hematoxylin and eosin (H&E).

Immunohistochemical staining was performed on an automated Bond Max instrument (Leica Biosystems, Wetzlar, Germany) using the following primary antibodies: HHV‐8 (13B10, 1:40; LifeSpan Biosciences, Seattle, USA); IgG4 (MC011, 1:10 000; The Binding Site, Birmingham, UK); IgG (RWP49, 1:600; Novocastra, Newcastle, UK), CD20 (L26; 1:200; Novocastra, Newcastle, UK), and CD3 (PS1; 1:50; Novocastra, Newcastle, UK). In situ hybridization was also performed for the κ and λ light chains (Leica Biosystems, Wetzlar, Germany).

The number of IgG‐ and IgG4‐positive cells was estimated in the area with the highest density of IgG4‐positive cells. The numbers of cells in three different HPFs (eyepiece, 10×, and lens, 40×) were counted, and the average was calculated to obtain the number of IgG4‐positive cells and the IgG4‐/IgG‐positive cell ratio.

### Validation study

We applied the pathological and comprehensive criteria^5^, ^12^ to 58 PC‐iMCD cases (39 lymph node lesions and 19 lung lesions); eight cases each of lymph node and lung lesions met the criteria. For these 16 cases, the IgG4‐RD exclusion criteria^10^ were applied, and diagnostic accuracy was determined on the basis of the number of cases that met any one of the clinical and pathological criteria (“continuing elevated serum level of C‐reactive protein [CRP],” “elevated serum level of IgA,” and “elevated serum level of IgM,” “sheet‐like proliferation pattern of mature PCs,” “high degree of hemosiderin deposition,” and “neutrophilic infiltration”).

### Analysis of clinical data and IgG4‐positive cells

Clinical information, including age, gender, and laboratory data (CRP, immunoglobulins [IgG, Ig4, IgA, IgM, and IgE]) was obtained from medical records. The correlation between the number of IgG4‐positive cells in tissues and the serum immunoglobulin levels was examined and compared between PC‐iMCD and IgG4‐RD.

### Statistical analyses

Statistical analyses were conducted using SPSS for Windows version 23.0 (SPSS, Chicago, IL, USA). A *p* < 0.05 was considered statistically significant.

## RESULTS

### Laboratory findings

The demographics and laboratory findings of the patients are summarized in Table 2.

**Table 2:** Comparison of clinical findings between PC‐iMCD and IgG4‐RD

	Lymph node			Lung		
	PC‐iMCD (*n* = 39)	IgG4‐RD (*n* = 22)	*p*‐value	PC‐iMCD (*n* = 19)	IgG4‐RD (*n* = 7)	*p*‐value
Age (mean ± SD)	53.9 ± 13.1	68.1 ± 9.3	**<0.001**	49.0 ± 11.5	65.9 ± 12.5	**0.008**
Sex (M/F)	25/14	7/15	‐	8/11	7/0	‐
**Laboratory data (mean** ± **SD)**						
IgG (mg/dL)	4677 ± 1655^a^	3393 ± 1309^b^	**0.013**	4250 ± 1423^c^	2770 ± 1192	**0.018**
IgG4 (mg/dL)	612 ± 670^a^	836 ± 659^b^	0.335	573 ± 448^c^	1142 ± 590	**0.013**
serum IgG4/IgG (%)	12.3 ± 9.3^a^	31.6 ± 10.7^b^	**<0.001**	12.8 ± 8.9^c^	40.7 ± 12.8	**<0.001**
IgA (mg/dL)	562 ± 251^a^	152 ± 58^b^	**<0.001**	599 ± 318^c^	193 ± 75^d^	**<0.001**
IgM (mg/dL)	240 ± 152^a^	66 ± 41^b^	**<0.001**	369 ± 89^c^	ND	‐
IgE (IU/mL)	2929 ± 3760^a^	878 ± 798^b^	0.202	1149 ± 846^c^	1812 ± 1800^d^	1.000
CRP (mg/dL)	6.0 ± 4.6^a^	0.1 ± 0.1^b^	**<0.001**	4.6 ± 3.4^c^	0.5 ± 0.6	**<0.001**

*Note*: Significant *p*‐values are in bold. Significance was calculated using the Mann–Whitney *U*‐test. Normal ranges: IgG, 870–1700 mg/dl; IgG4, 4.8–105 mg/dl; IgA, 110–410 mg/dl; IgM, 31–200 mg/dl (male), 52–270 mg/dl (female); IgE, 0–170 IU/ml; CRP, 0.00–0.30 mg/dl.

Abbreviations: CRP, C‐reactive protein; Ig, immunoglobulin; IgG4‐RD, immunoglobulin G4‐related disease; PC‐iMCD, plasma cell type idiopathic multicentric Castleman disease; ND, not done; SD, standard deviation.

^a^ IgG, IgG4, serum IgG4/IgG, IgA, IgM, IgE, and CRP were available for 33, 26, 18, 19, 19, 7, and 31 patients, respectively.

^b^ IgG, IgG4, serum IgG4/IgG, IgA, IgM, IgE, and CRP were available for 15, 17, 12, 12, 12, 5, and 18 patients, respectively.

^c^ IgG, IgG4, serum IgG4/IgG, IgA, IgM, IgE, and CRP were available for 19, 17, 17, 16, 2, 13, and 19 patients, respectively.

^d^ IgA and IgE levels were available for 6 and 5 patients, respectively.

### Findings in lymph node lesions

Among the patients with lymph node lesions, 25 males and 14 females were diagnosed with PC‐iMCD, whereas seven males and 15 females were diagnosed with IgG4‐RD. The mean ages of the PC‐iMCD and IgG4‐RD patients were 53.9 (range 34–89 years) and 68.1 years (range 47–81 years), respectively. PC‐iMCD patients were younger than IgG4‐RD patients (*p* < 0.001).

The laboratory findings revealed that the levels of serum IgG, IgA, IgM, and CRP in the PC‐iMCD patients were significantly higher than those in the IgG4‐RD patients (*p* = 0.013, *p* < 0.001, *p* < 0.001, and *p* < 0.001, respectively). The serum IgG4/IgG ratio of IgG4‐RD patients was higher than that of PC‐iMCD patients (*p* < 0.001). There was no significant difference in serum IgG4 and serum IgE levels between the two groups (*p* = 0.335 and *p* = 0.202, respectively).

Further, 15/18 (83.3%) patients with PC‐iMCD had serum IgG4 levels >135 mg/dL. Elevation of IgA and IgM above the reference level was observed in 15/19 (78.9%) and 11/19 (57.9%) of PC‐iMCD patients, respectively, while none of them was observed in IgG4‐RD patients. Elevation of CRP ≥ 1.0 mg/dL was observed in all patients with PC‐iMCD (31/31) but not in patients with IgG4‐RD (0/18).

### Findings in lung lesions

Among patients with lung lesions, 8 males and 11 females were diagnosed with PC‐iMCD, whereas seven males were diagnosed with IgG4‐RD. The mean ages of the PC‐iMCD and IgG4‐RD patients were 49 (range 26–73 years) and 65.9 years (range 39–77 years), respectively. PC‐iMCD patients were younger than IgG4‐RD patients (*p* = 0.008).

The laboratory data indicated that the levels of serum IgG, IgA, and CRP were significantly higher in the PC‐iMCD group than in the IgG4‐RD group (*p* = 0.018, *p* < 0.001, *p* < 0.001, respectively). The serum IgG4 levels and IgG4/IgG ratio of the IgG4‐RD group were significantly higher than those in the PC‐iMCD group (*p* = 0.013 and *p* < 0.001, respectively). There was no significant difference between the serum IgE levels of the two groups (*p* = 1.000).

All cases (17/17) with PC‐iMCD had serum IgG4 levels >135 mg/dL. Elevation of IgA and IgM above the reference level was observed in 12/16 (75.0%) and 2/2 (100%) of PC‐iMCD patients, respectively, while none of them was observed in IgG4‐RD patients. Elevation of CRP ≥ 1.0 mg/dL was observed in 17/19 of PC‐iMCD patients and one IgG4‐RD patient (1/7).

### Pathological and immunohistochemical findings

**Figure 1:**
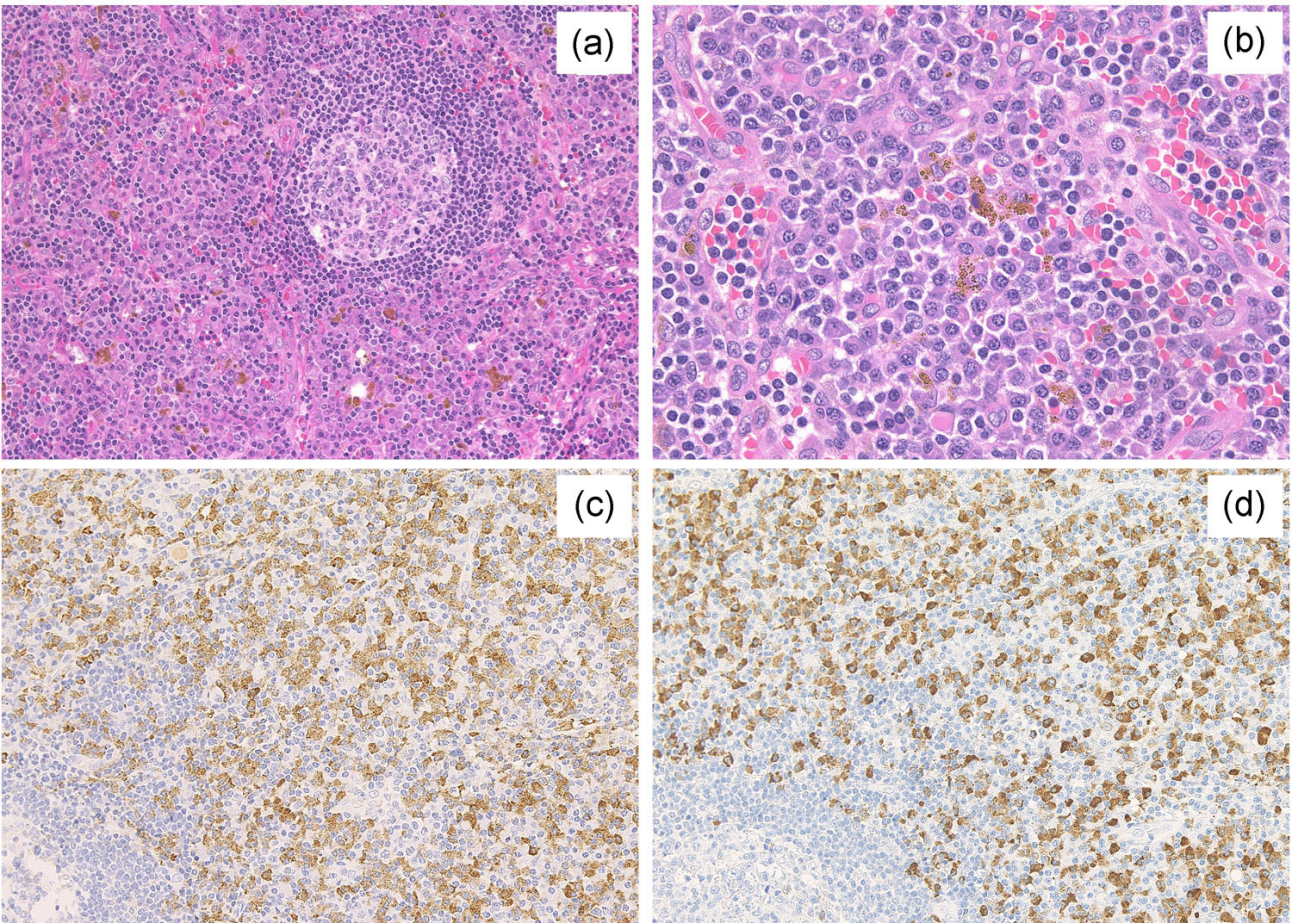
Pathological features of PC‐iMCD patients in lymph nodes. Expansion of the interfollicular area with moderately vascular proliferation and infiltration of abundant plasma cells were observed (a, HE ×100). Sheet‐like proliferation of mature plasma cells and high degree of hemosiderin deposition was observed in interfollicular area (b, HE × 400). The average number of IgG4‐positive cells was >400/HPF, and IgG4/IgG‐positive cell ratio was 93.5% (c, IgG × 200; d, IgG4 × 200). HE, hematoxylin and eosin; HPF, high‐power field; Ig, immunoglobulin; PC‐iMCD, plasma cell idiopathic multicentric Castleman disease

Pathologically, the lymph node and lung lesions of PC‐iMCD patients exhibited normal to hyperplastic germinal centers with expanded interfollicular area. Sheet‐like proliferation of mature PCs and hemosiderin deposition were observed in the lymph node from PC‐iMCD patients (Figure 1). In contrast, the lymph node and lung lesions of IgG4‐RD patients exhibited mixed infiltration of PCs, eosinophils, small lymphocytes, and immunoblasts.

A large number of IgG4‐positive cells were found in both the lungs and lymph nodes of PC‐iMCD patients. The number of IgG4‐positive cells and IgG4/IgG‐positive cell ratio in PC‐iMCD patients are shown in Table 3. In PC‐iMCD, the mean number of IgG4‐positive cells was 124/HPF in lymph node lesions (range 17–401 cells) and 103/HPF in lung lesions (range 3–223 cells). IgG4‐positive cells with >50/HPF were identified in 35 of the lymph node lesions (89.7%) and 15 of the lung lesions (78.9%). Additionally, there were 21 lymph node lesions (53.8%) and nine lung lesions (47.4%) with IgG4‐positive cells exhibiting >100/HPF.

**Table 3:** The number of IgG4‐positive cells and IgG4/IgG ratio in PC‐iMCD

	Lymph node (*n* = 39)	Lung (*n* = 19)
The number of IgG4‐positive cell/HPF (mean ± SD)	124 ± 87	103 ± 64
>10/HPF, *n* (%)	39 (100)	18 (94.7)
>50/HPF, *n* (%)	35 (89.7)	15 (78.9)
>100/HPF, *n* (%)	21 (53.8)	9 (47.4)
IgG4/IgG ratio (mean ± SD)	31.6 ± 20.8	43.0 ± 25.8
IgG4/IgG ratio >40%, *n* (%)	8 (20.5)	8 (42.1)
>50/HPF and IgG4/IgG ratio >40%, *n* (%)	8 (20.5)	8 (42.1)
>100/HPF and IgG4/IgG ratio >40%, *n* (%)	8 (20.5)	7 (36.8)

*Note*: The number of IgG4‐positive cells (/HPF) is the average of three different HPFs with the highest density.

Abbreviations: HPF, high‐power field; Ig, immunoglobulin; PC‐iMCD, plasma cell type idiopathic multicentric Castleman disease.

Eight cases of lymph node lesions (20.5%) and eight cases of lung lesions (42.1%) met the pathological criteria for IgG4‐RD (IgG4‐positive cell with >50/HPF and IgG4/IgG ratio >40%).

### Validation of exclusion criteria for IgG4‐RD

The clinical and pathological findings of 16 PC‐iMCD patients who met the pathological criteria for IgG4‐RD^5^ are summarized in Table 4. Among these patients, 7/8 cases (87.5%) with lymph node lesion and 6/8 cases (75.0%) with lung lesion had elevated CRP levels (≥1.0 mg/dL). Serum IgA levels were elevated in three lymph node cases and five lung cases in which IgA was measurable. Serum IgM levels were elevated in all cases, except one case with lymph node lesion.

**Table 4:** Application of exclusion criteria 2020 to IgG4‐RD and PC‐iMCD that met the diagnostic criteria for IgG4‐RD

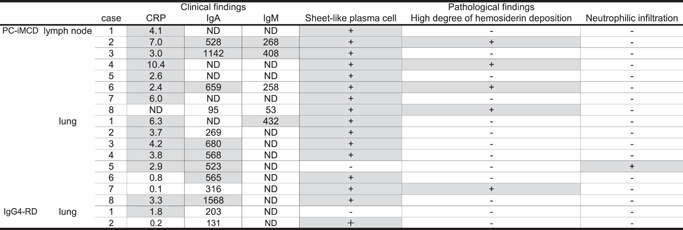

*Note*: Normal ranges: CRP, 0.00–0.30 mg/dl; IgA, 110–410 mg/dl; IgM, 31–200 mg/dl (male), 52–270 mg/dl (female). The gray background indicated, which is met the exclusion criteria.

Abbreviations: CRP, C‐reactive protein; Ig, immunoglobulin; IgG4‐RD, immunoglobulin G4‐related disease; ND, not done; PC‐iMCD, plasma cell type idiopathic multicentric Castleman disease.

Pathological findings revealed sheet‐like proliferation of mature PCs in all cases, except one case with lung lesion. A high degree of hemosiderin deposition was observed in 4/8 (50%) cases with lymph node lesion and 1/8 (12.5%) case with lung lesion. Neutrophilic infiltration was observed in one lung lesion.

**Figure 2:**
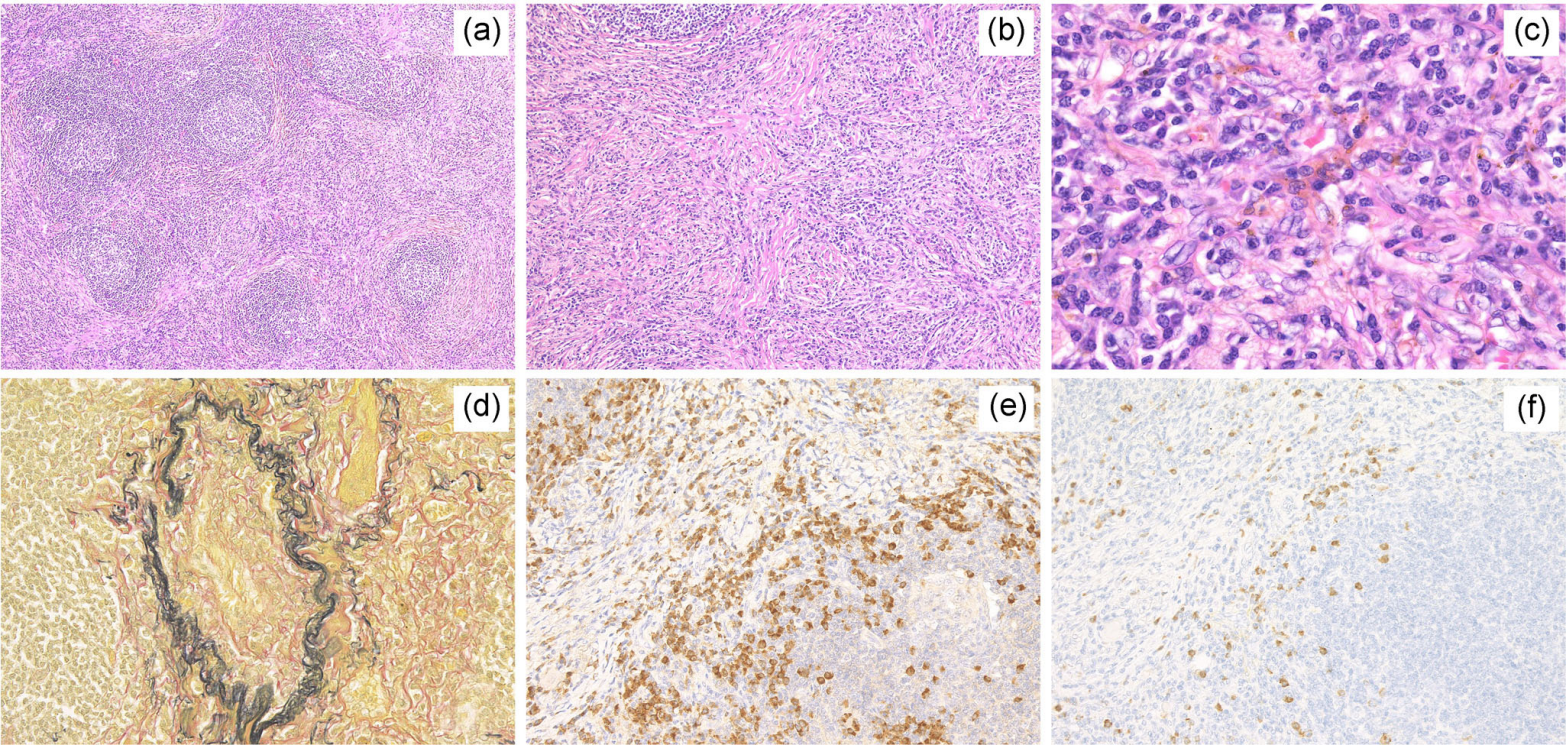
Pathological features of pulmonary lesions in a patient with PC‐iMCD showing storiform fibrosis and obliterative vasculitis. Expanded Interfollicular area and mild fibrosis were observed (a, HE × 40). Storiform fibrosis (b, HE × 100) was seen. Marked hemosiderin deposition was observed (c, HE × 400). Obliterative vasculitis (d, Elastica‐van Gieson, × 200) was seen. The average number of IgG4‐positive cells was >100/HPF, and IgG4/IgG‐positive cell ratio was 27.6% (e, IgG × 200; f, IgG4 × 200). HE, hematoxylin and eosin; HPF, high‐power field; Ig, immunoglobulin; PC‐iMCD, plasma cell idiopathic multicentric Castleman disease

In lung lesions of PC‐iMCD patients, obliterative vasculitis was sometimes observed (12/19, 63.2%). In one PC‐iMCD case with lung lesion, storiform‐like fibrosis and obliterative vasculitis were observed, which were difficult to differentiate from those in IgG4‐RD. However, in this case, marked hemosiderin deposition was observed, and the criterion of IgG4/IgG‐positive cell ratio >40% was not met (Figure 2).

As a result, all 16 patients who met the pathological criteria for IgG4‐RD were excluded by the exclusion criteria.^10^

There were no IgG4‐RD cases with lymph node lesions that met the exclusion criteria for IgG4‐RD.^10^ However, in the lung lesions of IgG4‐RD patients, one case had a slightly elevated CRP level (1.8 mg/dL), and another had a sheet‐like proliferation of mature PCs. The results showed that 2/29 (6.9%) IgG4‐RD cases met the exclusion criteria.^10^ Based on the combined clinical and pathological findings, these cases were finally diagnosed as IgG4‐RD.

Thus, the sensitivity and specificity of the exclusion criteria^10^ were 100% and 93.1%, respectively.

### Relationship between serum data and IgG4‐positive cells

**Figure 3:**
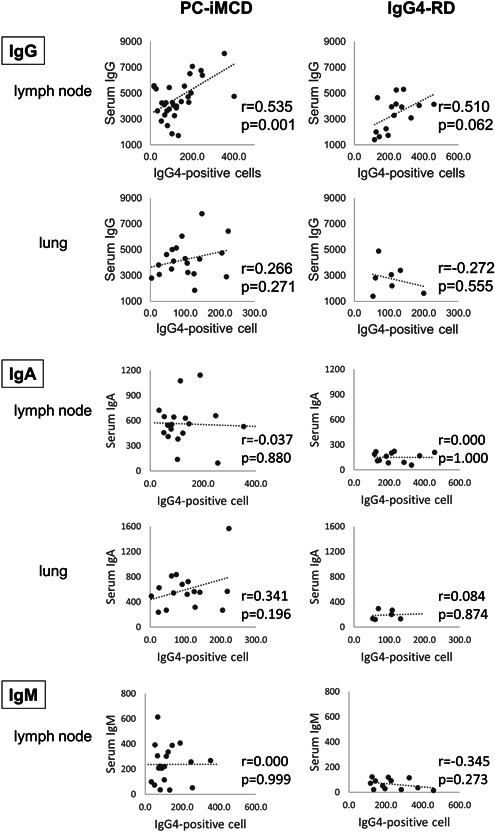
Relationship between the number of IgG4‐positive cells and serum data in PC‐iMCD and IgG4‐RD. Correlation between the number of IgG4‐positive cells and serum IgG, IgA, IgM in PC‐iMCD, and IgG4‐RD. The positive correlation was only between serum IgG level and the number of IgG4‐positive cells (/HPF) in lymph node lesions of PC‐iMCD (*p* = 0.001). HPF, high‐power field; Ig, immunoglobulin; IgG4‐RD, immunoglobulin G4‐related disease; LN, lymph node; PC‐iMCD, plasma cell idiopathic multicentric Castleman disease

The relationship between the number of IgG4‐positive cells and serum immunoglobulins is illustrated in Figure 3.

In the lymph node cases, there was a significant positive correlation between the serum IgG levels and the number of IgG4‐positive cells in PC‐iMCD group (*r* = 0.535, *p* = 0.001). Positive correlations were also noted between serum IgG levels and the number of IgG4‐positive cells in lymph node cases in the IgG4‐RD group and lung lesion cases in the PC‐iMCD group, but these correlations were not statistically significant (*r* = 0.510 and *p* = 0.062, *r* = 0.266, and *p* = 0.271, respectively). A weak and insignificant inverse correlation was observed in the lung lesion cases of IgG4‐RD (*r* = −0.272, *p* = 0.555). Serum IgA and the number of IgG4‐positive cells showed no relationship in lymph node cases in both the PC‐iMCD and IgG4‐RD groups. In contrast, there was a weak positive correlation in lung lesion cases of PC‐iMCD (*r* = 0.341, *p* = 0.196). Serum IgM and the number of IgG4‐positive cells were not correlated in the lymph node cases of PC‐iMCD. The IgM levels in lung lesion cases were not evaluated because they were rarely measured.

## DISCUSSION

Plasma cell type idiopathic multicentric Castleman disease is a rare lymphoproliferative disorder characterized by sheet‐like proliferation of mature PCs and systemic inflammatory symptoms.^13^ Although the clinicopathological features of IgG4‐RD partially overlap with those of PC‐iMCD,^14^, ^15^, ^16^ the clinical characteristics and treatment procedures for these diseases are different. Therefore, it is important for clinicians and pathologists to avoid misdiagnosing mimickers, such as PC‐iMCD, as IgG4‐RD.^17^

Although the etiology of IgG4‐RD remains unclear, an immune response involving T‐helper cell 2 (Th2) and regulatory T cells (Treg) is thought to play an important role. Upregulation of Th2 cytokines, such as IL‐4, IL‐5, and IL‐13, promotes the production of IgE and eosinophils.^18^ Further, IL‐4 expression in B cells promotes IgG4 production. Treg cytokines, such as transforming growth factor (TGF) β and IL‐10, are also thought to be involved in fibrosis formation and IgG4 production.^7^, ^19^

Overproduction of IL‐6 is deeply related to PC‐iMCD pathogenesis.^20^ IL‐6 is produced by a variety of cells, including T cells, B cells, and macrophages. This pro‐inflammatory cytokine activates B cells and promotes their maturation into PCs that produce immunoglobulins.^20^ This leads to a polyclonal increase in immunoglobulins, resulting in elevated serum levels of all subclasses, including IgG4.^21^, ^22^ Moreover, increased serum IL‐6 concentration has also been associated with other serological abnormalities, such as elevated serum CRP^6^ and thrombocytosis, as IL‐6 is a major stimulatory factor for hepatocytes and multipotent hematopoietic progenitors.^20^ Excessive production of IL‐6 is also closely associated with hemosiderin deposition.^23^ Dysregulated production of hepcidin has been linked to anemia and inflammation, and IL‐6 is a major inducer of hepcidin production. Hepcidin reduces intestinal iron absorption and blocks iron release from macrophages, resulting in hemosiderin deposition in the lesion.^23^

Using the differences in pathogenesis,^24^ exclusion criteria for IgG4‐RD were proposed in 2020 to accurately differentiate between IgG4‐RD and its mimic, PC‐iMCD.

In PC‐iMCD, a higher number of IgG4‐positive cells in the tissue is more likely cause it to be misdiagnosed as IgG4‐RD. Since the exclusion criteria include serum IgA and IgM levels, we were interested in the relationship between the number of IgG4‐positive cells in the tissues and serum gamma globulin levels and investigated the correlation.

In the current analysis, a positive correlation was noted between the number of IgG4‐positive cells and serum IgG levels in both lymph nodes and lungs of PC‐iMCD, but a significant difference was only seen in lymph nodes. This result may be related to the number of affected lesions. Most of our cases in which lymph node lesions of PC‐iMCD showed multiple lymphadenopathy, whereas in the group of cases in which lung lesions were sampled, lesions other than lung were localized to a few sites. This may mean that the serum IgG level is related to the overall amount of lesions. If this hypothesis is correct, serum IgG level and number of IgG4‐positive cells may reflect the disease activity of PC‐iMCD, but further study is needed.

In this study, all PC‐iMCD cases that met the pathological criteria for IgG4‐RD^5^ were effectively excluded using the exclusion criteria.^10^ If at least one of the clinical or pathological exclusion criteria is identified, it is important to reconsider the diagnosis, even if the diagnostic criteria for IgG4‐RD have been met. In such cases, it is essential to rule out the possibility of hyper IL‐6 syndrome, including PC‐iMCD, before confirming the diagnosis of IgG4‐RD. As our previous reports have shown,^11^, ^21^ immunohistochemical staining for IgA and IL‐6 may be useful in making the diagnosis of hyper IL‐6 syndrome in this situation.

In IgG4‐RD, there were two cases that met the exclusion criteria. In one of the cases, dense proliferation of mature PCs was observed, but there was also a prominent eosinophilic and lymphocytic infiltration (Figure S1), which was more suggestive of IgG4‐RD.^11^ In addition, both cases showed a characteristic distribution of lesions in IgG4‐RD (lacrimal and salivary glands in one case and pancreatic lesions in the other), and steroid administration resulted in rapid improvement of symptoms and laboratory data, indicating that these cases should have been diagnosed as IgG4‐RD. Note that if the exclusion criteria are met, it does not mean that the possibility of IgG4‐RD is completely ruled out. It is important to make an accurate diagnosis based on the combination of clinical symptoms (including affected organ distribution) and other pathological findings, such as eosinophilic infiltration and immunostaining for IgA and IL‐6.^11^, ^21^ In addition, the evaluation of sheet‐like proliferation of mature PCs can vary among pathologists, and the evaluation method could be improved in the future.

In conclusion, we validated the accuracy of the proposed exclusion criteria for IgG4‐RD^10^ in PC‐iMCD cases. The proposed IgG4‐RD exclusion criteria^10^ demonstrated a sensitivity of 100% and specificity of 93.1%, indicating that these exclusion criteria are useful for both pathologists and clinicians to avoid misdiagnosis.

## DISCLOSURE

The authors declare no competing interests.

## AUTHOR CONTRIBUTIONS

*Conception and design of the study*: Asami Nishikori, Midori Filiz Nishimura, and Yasuharu Sato. *Acquisition and analysis of data*: Asami Nishikori and Midori Filiz Nishimura. *Drafting the manuscript*; Asami Nishikori and Midori Filiz Nishimura. *Review and editing the manuscript*: Yoshito Nishimura, Kenji Notohara, Akira Satou, and Seiji Nakamura. *Resources*; Yasuharu Sato. *Supervision*; Midori Filiz Nishimura and Yasuharu Sato.

## Supporting information


**Supplementary figure 1**.
