# Supplementary figures and images for: Investigation of IgG4‐positive cells in idiopathic multicentric Castleman disease and validation of the 2020 exclusion criteria for IgG4‐related disease
Source: Pathol Int. 2021 Nov 11;72(1):43–52. doi: 10.1111/pin.13185 (PMC9299129; doi:10.1111/pin.13185)

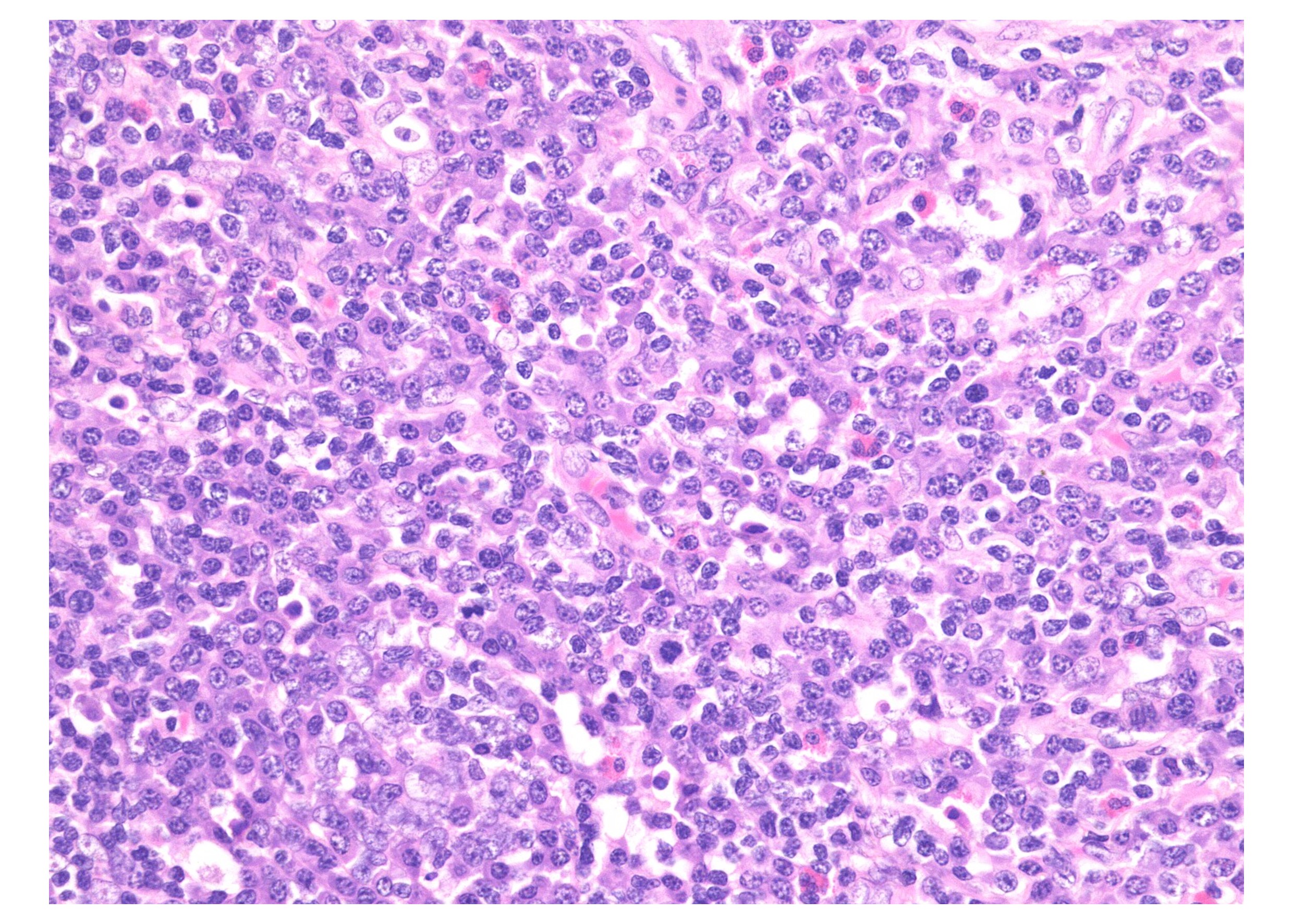

Supplement: Supplementary file 1 — Supplementary figure 1. [file PIN-72-43-s001.jpg]
